# Non-drinking and social life in adolescence: a qualitative study in Switzerland

**DOI:** 10.1007/s44155-024-00124-x

**Published:** 2024-11-19

**Authors:** Lorraine Chok, Joan-Carles Suris, Lucie Vittoz, Diana Fernandes Palhares, Yara Barrense-Dias

**Affiliations:** Department of Epidemiology and Health Systems, Research Group On Adolescent Health, Center for Primary Care and Public Health (Unisanté), University of Lausanne, Route de La Corniche 10, 1010 Lausanne, CH Switzerland

**Keywords:** Abstinence, Alcohol consumption, Non-drinkers, Social life, Youth

## Abstract

**Background:**

Alcohol consumption is popular among adolescents and young people and adolescent non-drinkers may be socially excluded and/or stigmatized. The aim of this qualitative study was to explore the social life of young non-drinkers (14–20 years old), to understand how they live their non-drinking and how they are perceived by their drinking peers.

**Methods:**

We conducted a qualitative research on non-consumption of alcohol in the French-speaking region of Switzerland. Our study included 63 young people divided into 12 focus groups aged 14 to 20 years.

**Results:**

Overall, young non-drinkers would be a minority in their peer groups, leading some of them to feel out of the norm. Participants reported that not drinking alcohol is generally questioned and non-drinkers are almost always asked to justify their non-consumption. Finally, non-drinkers are sometimes automatically designated as resources who have to control the drinking of others and/or are the ones who help their drinking-peers if they are drunk and need help, a status that can put them under pressure.

**Conclusions:**

It is important to normalize the non-consumption of alcohol and make non-drinkers visible by including them in school-based prevention interventions.

## Background

Alcohol non-consumption among adolescents has increased in the past years in the Western world [1–7]. For instance, according to the European School Project on Alcohol and Drugs (ESPAD), there has been a slight decrease in alcohol consumption among 15–16 year-olds between 2003 and 2015 [8]. Another study led among 18–22 year-olds in the US found an increase in non-consumption of alcohol between 2002 and 2018[9]. In Switzerland, the same trend has been observed in the Health Behaviour in School-aged Children (HBSC) study [10] showing that weekly drinking prevalence rates decreased between 1986 and 2018 among 15-year-olds. Finally, in the Swiss Health Survey, the percentage of non-drinkers among 15–24 year-olds males and females increased respectively from 16.6% and 20.4% in 2012 to 21.1% and 27.8% in 2017 [11]. In a study led among 12–19-year-olds in England [12], the reasons for this decline could be the cost of alcohol, a new culture where alcohol would no longer be highly valued, awareness of risks associated with alcohol consumption, a stricter regulatory environment, a greater parental surveillance than before or the replacement of alcohol by other substances. In another study led among 18–25-year-olds in England [13], positive effects on physical and psychological health were one of the main benefits of not drinking reported by the participants. However, this would not always be enough motivation not to drink in the face of the social disadvantage that non-drinkers might feel. Following Pennay et al. [14], reasons may be different according to gender. For example, males might be more likely to report sports and females healthy lifestyle as a reason for light or non-drinking. Finally, this decline in alcohol use could be explained, according to some authors, by a change in ‘doing’ masculinity [14–16] where masculinity, according to gender norms, can be powerfully performed in another way than alcohol intoxication, such as doing sport.

However, alcohol consumption remains popular among adolescents and young people [8]. In drinking cultures, alcohol use is a way to socialize and build friendships [17]. In such contexts, adolescents’ non-drinkers may be socially excluded and/or stigmatized [6, 18, 19]. For instance, 18–29 year-old non-drinkers reported in a qualitative study led in Finland and Australia [6] that non-drinking alcohol could affect their social life. Indeed, some of them would avoid events with alcohol or would not be invited to parties. To manage their choice not to drink, adolescents and young people may develop strategies such as stop attending events associated with alcohol use, get together with non-drinking friends only, join groups where the culture is to abstain from substances and giving legitimate reasons such as driving [6, 20].

According to the World Health Organization, the European Region has the highest alcohol consumption in the world [21]. Switzerland, which has a strong drinking culture through its viticulture [22], is not an exception. Given the raising prevalence of abstinence among young people, there is a need to better understand how young non-drinkers live their abstinence in relation to their drinking peers [6] and more particularly if they experience stigma, especially in a drinking context such as Switzerland.

The aims of this qualitative study were to explore the social life of young non-drinkers in the French-speaking part of Switzerland (mean age 16.9 years) and to understand how they live their abstinence in relation to their drinking peers.

## Methods

We performed 12 focus groups (FG) with young people (drinkers and non-drinkers; females and males separately) aged between 14 and 20 between April and June 2022. The sample was composed of 29 drinkers (55.2% of females) with a mean and median age of 17, and 34 non-drinkers (58.8% of females) with a mean age of 16.8 and a median age of 16. No variation in terms of age regarding alcohol use status (e.g. mean age was 16.8 for non-drinkers and 17 for drinkers) and gender (e.g. mean age was 16.9 for both genders) were found. The interactivity between participants induced by FGs allow them to share and defend their points of view. FGs are then particularly of interest to explore an understudied phenomenon [23].

### Procedure

Our study is reported following the Standards for Reporting Qualitative Research (SRQR) [24] and we used the Consolidated criteria for reporting qualitative research (COREQ) checklist [25] to report the important aspects of our research in this article. Focus groups were conducted by two female researchers (Y.B-D. and L.C). The first one was conducted in a public health center, in a room dedicated to the FG. Participants came directly and did not need to announce themselves in advance, at a reception for example. The other discussions were conducted by video conference using the secure professional version of the Zoom© platform due to the sanitary measures imposed by the COVID-19 pandemic, and because of difficulties in recruiting young people for discussions in person, perhaps due to COVID habits. The participants were mainly recruited through sponsored ads on the social media Instagram©, adolescent-oriented websites, offline postings and, occasionally, through a snowball method. The initial advertisement called for young people aged between 14 and 20 years old who drank alcohol or not and were interested in talking about this topic. Every two weeks, we readjusted the campaign on this social network in order to reach an audience according to the responses we had received so far (for example, targeting males or 14–15 year olds). When adolescents contacted us, we inquired about their gender, age and drinking status by asking them which of the following groups they belonged to: I have never drunk alcohol/I used to drink alcohol but I stopped/I sometimes drink alcohol but I have never been drunk./I sometimes drink alcohol and I have already been drunk.

### Instrumentation

Each FG lasted about 1 h and was moderated by Y.B-D. and L.C. and an observer (L.C. or J-C.S.) who took notes. Prior to each FG all participants received an online socio-demographic questionnaire on the secure REDCap® application, which also included an information sheet about the study and signed an electronic consent form. Every recording was anonymously transcribed verbatim and then deleted. To ensure anonymity, all identification elements (first name, surname, address, etc.) were removed in the transcriptions. All the details of the recording including the transcription and anonymization were presented to the participants. A semi-structured focus group guide based on the literature, quantitative results of a previous study [26] and discussions with experts in the field was created. It included questions such as “What does ‘not drinking alcohol’ mean to you as a young person?”, “What does ‘abstinence’ related to alcohol mean to you as a young person?”, “What is your opinion about young people who don't drink?” Why abstaining from alcohol?” “As a young person, is there a pressure to drink?”, “How do you perceive this pressure?”, “What are the advantages of not drinking?” and “Does it have an effect on social life?”. At the end of each FG, participants received a 30 Swiss Francs (∼30 US$) gift card for a large department store as a way to thank them for their time. The protocol was validated by the Cantonal Ethics Committee (protocol #2022-00063). A safety net was intended for problematic cases that might come to the knowledge of the authors. Adolescents with personal difficulties could be referred to support institutions and care units if needed. This information was included in the information letter. A detailed description of the survey methodology can be found elsewhere [19].

### Participants

Participants inclusion criteria were to be aged between 14 and 20 years and fluent in French. A total of 63 participants took part in the study. We chose to separate participants by gender as a difference in alcohol consumption is often demonstrated [10, 27, 28]. Moreover, gender homogeneity is often recommended when FG are conducted with young people, thus avoiding as much as possible an adaptation of their speech or embarrassment about gender differences [29, 30]. We also separated participants into three groups according to their age (14–15, 16–17 and 18–20-year-olds) because legal drinking age is 16 for wine and beer and 18 for spirits in Switzerland. Finally, we separated drinkers and non-drinkers since their experiences and perceptions of alcohol may be different. Therefore, adolescents (mean age 16.9 years old and median 16 years) were separated by age, gender and drinking status (Table 1). No adolescent of any other gender category contacted us.

**Table 1:** Sociodemographic data and drinking status of the participants

	Gender	Age	Drinking status	Activity^a^
Focus Group 1 (N = 5)—Female drinkers under 18				
Participant 1	Female	16	Drinker	Post-mandatory (2nd)
Participant 2	Female	16	Drinker	Post-mandatory (2nd)
Participant 3	Female	16	Drinker	Vocational track (1st)
Participant 4	Female	16	Drinker	Post-mandatory (2nd)
Participant 5	Female	17	Drinker	Post-mandatory (2nd)
Focus Group 2 (N = 7) Female drinkers 18 or older				
Participant 6	Female	19	Drinker	University (1st)
Participant 7	Female	18	Drinker	Post-mandatory (3rd)
Participant 8	Female	20	Drinker	Post-mandatory (4th)
Participant 9	Female	20	Drinker	University (1st)
Participant 10	Female	18	Drinker	Post-mandatory (3rd)
Participant 11	Female	20	Drinker	University (2nd)
Participant 12	Female	19	Drinker	University (1st)
Focus Group 3 (N = 4) Male drinkers 18 or older				
Participant 13	Male	18	Drinker	Sabbatical year
Participant 14	Male	20	Drinker	University (1st)
Participant 15	Male	19	Drinker	University (1st)
Participant 16	Male	20	Drinker	Without activity
Focus Group 4 (N = 7) Female non-drinkers 18 or older				
Participant 17	Female	20	Non-Drinker	Bridging program^b^
Participant 18	Female	19	Non-Drinker	Post-mandatory (4th)
Participant 19	Female	20	Non-Drinker	University (1st)
Participant 20	Female	19	Non-Drinker	University (1st)
Participant 21	Female	19	Non-Drinker	Working
Participant 22	Female	18	Non-Drinker	Post-mandatory (3rd)
Participant 23	Female	18	Non-Drinker	Transition school^c^
Focus Group 5 (N = 4) Male non-drinkers under 18				
Participant 24	Male	16	Non-Drinker	Vocational track
Participant 25	Male	16	Non-Drinker	Vocational track
Participant 26	Male	16	Non-Drinker	Post-mandatory (1st)
Participant 27	Male	17	Non-Drinker	Post-mandatory (2nd)
Focus Group 6 (N = 4) Female drinkers under 18				
Participant 28	Female	15	Drinker	Mandatoriy school (11H)
Participant 29	Female	15	Drinker	Mandatory school (11H)
Participant 30	Female	15	Drinker	Mandatory school (11H)
Participant 31	Female	14	Drinker	Mandatory school (11H)
Focus Group 7 (N = 6) Female non-drinkers under 18				
Participant 32	Female	16	Non-Drinker	Post-mandatory (2nd)
Participant 33	Female	17	Non-Drinker	Post-mandatory (2nd)
Participant 34	Female	17	Non-Drinker	Post-mandatory (2nd)
Participant 35	Female	16	Non-Drinker	Mandatory school (11H)
Participant 36	Female	16	Non-Drinker	Post-mandatory (2nd)
Participant 37	Female	17	Non-Drinker	Post-mandatory (1st)
Focus Group 8 (N = 5) Male drinkers under 18				
Participant 38	Male	17	Drinker	Post-mandatory (3rd)
Participant 39	Male	17	Drinker	Post-mandatory (3rd)
Participant 40	Male	17	Drinker	Post-mandatory (2nd)
Participant 41	Male	16	Drinker	Mandatory school (11H)
Participant 42	Male	16	Drinker	Post-mandatory (1st)
Focus Group 9 (N = 4) Male drinkers under 18				
Participant 43	Male	15	Drinker	Mandatory school (11H)
Participant 44	Male	15	Drinker	Post-mandatory (2nd)
Participant 45	Male	15	Drinker	Mandatory school (11H)
Participant 46	Male	15	Drinker	Mandatory school (11H)
Focus Group 10 (N = 7) Female non-drinkers under 18				
Participant 47	Female	15	Non-Drinker	Mandatory school (11H)
Participant 48	Female	14	Non-Drinker	Mandatory school (11H)
Participant 49	Female	15	Non-Drinker	Mandatory school (11H)
Participant 50	Female	15	Non-Drinker	Mandatory school (11H)
Participant 51	Female	15	Non-Drinker	Mandatory school (10H)
Participant 52	Female	15	Non-Drinker	Mandatory school (11H)
Participant 53	Female	15	Non-Drinker	Mandatory school (11H)
Focus Group 11 (N = 5) Male non-drinkers 18 or older				
Participant 54	Male	18	Non-Drinker	Post-mandatory (2nd)
Participant 55	Male	19	Non-Drinker	Post-mandatory (2nd)
Participant 56	Male	20	Non-Drinker	Post-mandatory (3rd)
Participant 57	Male	20	Non-Drinker	University (1st)
Participant 58	Male	20	Non-Drinker	Post-mandatory (3rd)
Focus Group 12 (N = 5) Male non-drinkers under 18				
Participant 59	Male	15	Non-Drinker	Mandatory school (11H)
Participant 60	Male	15	Non-Drinker	Mandatory school (11H)
Participant 61	Male	15	Non-Drinker	Mandatory school (11H)
Participant 62	Male	15	Non-Drinker	Mandatory school (11H)
Participant 63	Male	15	Non-Drinker	Mandatory school (11H)

^a^In Switzerland, school is mandatory until the age of around 15 (before high school)

^b^Bridging programs enable a student with a vocational or professional diploma to enter university

^c^Transition school is a structure for young people who have finished mandatory school but have not yet found a training/apprenticeship position

### Data analysis

FG transcripts were transferred to the software MAXQDA (version 20.0.8). An inductive approach was used to perform a thematic content analysis which enabled to extract the different themes and dimensions brought up by the participants [31–33]. Some codes were developed a priori with the FG grid and others emerged from the analysis. The extraction grid was tested on two discussions and then applied to code the other discussions by LC and YBD. The analyses were systematically crosschecked by JCS. Each new or different code was discussed between the authors. This method aims to focus on subjective interpretations and meanings of a social phenomenon by using a classification and categorization process. The analyses were carried out by YBD and LC and systematically reviewed by JCS to discuss any discrepancies. This process allowed for a form of triangulation at the level of the analysis, thus avoiding the risk of bias as much as possible. Quotes were translated from French into English.

## Results

Differences in terms of gender and age, when they emerged from the analysis, are expressly indicated.

### Reasons for non-drinking

Religion, legal ages, parents and family rules, bad experiences, distaste, religion, health consciousness and fear of losing control were the reasons reported by the participants for non-drinking. The only one which differed by gender were the fear of sexual violence which was reported by some female participants. Both females and males felt that females were at greater risk of sexual violence as a result of drinking. One female participant reported not drinking alcohol, specifically for this reason. Indeed, she reported already feeling vulnerable to sexual violence as a girl, and drinking alcohol would reinforce that vulnerability according to her. *“I do not drink, because we hear so many stories about girls being assaulted and so on. Actually, it is really fear […] when you are a girl, you are basically easy prey for the aggressors. So I say to myself ‘If you are drinking on top of that…’ ". (Female, 19, non-drinker).* Several participants felt that males generally did not have this fear of sexual violence that would impact their alcohol use. *“Generally, males do not have that ulterior motive to be careful about their alcohol use to stay safe." (Female, 20, drinker).* Finally, female participants mentioned the fear of having drugs put in their glasses. *"However, in relation to drinking, I find that being a female is much more risky than being a male. I mean, even drinking for pleasure can become dangerous, […] substances can be put in her glass […].” (Female, 17, non-drinker).*

### Friends and socialization

Overall, non-drinkers would be a minority in their peer groups, leading some of them to feel marginal. This was reported especially among older groups. *“Well, not drinking alcohol as a young person, there is also a bit of marginality. […] Those who drink alcohol in general are the majority at parties. […] There are maybe two or three per party (who do not drink alcohol).” (Female, 18, drinker).*

As a minority, some non-drinkers reported feeling out of the norm. For instance, a non-drinker participant referred to the term ‘alien’ to explain the way she feels to be perceived. This would happen especially as they get older and reach legal age with alcohol and partying becoming more normalized.

*“As soon as we get closer to the age of majority (18 years old in Switzerland), it is something normal (to drink alcohol) and if we do not, we are kind of weird or we are kind of seen as aliens.” (Female, 20, non-drinker).*

This normalization would also be felt in family contexts for some youth. For instance, some of them were surprised that they were naturally offered alcohol at the legal age when it was forbidden before and this fact was discussed among participants. *“[…] Up to a certain age, there is a ban on it (alcohol) […]. And what I found funny is that […] as soon as you reach 18 years or almost, you are immediately allowed to touch it.” (Female, 19, drinker).*

In terms of socialization, being in parties where people drink alcohol would not be a problem for some non-drinkers as long as their friends respect their choice.

*“I do not have any problem with being around people who drink. I mean, […] (the fact) that they drink does not mean that it is going to change anything socially. […] Most of the people I meet at parties drink and there are also people who have a lot of respect for whether or not we drink. For example, I have been to parties with friends where I did not drink at all and I was still included in the party […].” (Female, 17, non-drinker).*

However, some non-drinking participants reported that their social life was affected by their non-drinking status. For instance, they reported having ever been excluded from parties because they do not drink alcohol.

*“I think there are several parties where I was not invited to because I was not drinking, and there is a whole image around that.” (Male, 16, non-drinker).*

As a consequence, some non-drinkers would avoid parties where most people drink alcohol. University parties are one of those contexts that some non-drinkers would avoid because of the central place alcohol has. This has especially been reported among females. Other non-drinking participants, especially females, reported avoiding parties where most people drink alcohol because they feel uncomfortable in such contexts.

*“Do you personally feel that your social life is worse than that of young people who drink?” (Investigator). “I do, clearly, because in fact all the parties at the faculties and so on, I do not go, because I know there will be alcohol and it will not be an atmosphere in which I will feel good.” (Female, 19, non-drinker).*

Therefore, it may be difficult to participate to academic social life where partying and drinking are sometimes central to such events.

*“It is true that if we talk about […] the university world, it is difficult to meet the others. As long as the others do things that we do not really want to do, I mean, drinking alcohol, it is difficult to fit in. So on (academic) campus, yeah, social relations are quite complicated.” (Female, 20, non-drinker).*

### Comments and pressure

Discussions also focused on the comments that non-drinking youth might receive about not drinking. As mentioned above, some participants reported that comments were more frequent with age and alcohol use being legal, some young people considering that there was no longer a valid reason not to drink. *“At 16/17 years old you can still play the age card (when not drinking) […] and now they tell me ‘You are of age, it is okay, you can drink.’ […] Now I have no excuse.” (Male, 19, non-drinker).*

However, other participants considered that comments decreased with age and maturity. “*When I used to go to parties with my friends, yeah, it was quite frequent this kind of comments (pushing a young person to drink alcohol). But today, with the time that has passed, I think we have all matured a little […].” (Male, 18, drinker).*

It would therefore be easier for some young people to assert themselves as non-drinkers as growing up, particularly because the need to belong to a group would diminish. *“It is also the age (16 years old) when you need more (than about 18 years old) to belong to a group than to do what is good for you. […] You want to follow the group so much that you are ready to do anything.” (Female 19, non-drinker).*

However, overall, participants reported that not drinking alcohol was generally questioned and non-drinkers were almost always asked to justify their non-consumption. *"Every time I go to a party […], people ask me why I do not drink. But really, it is the question of the century: ‘What? You do not drink? I mean, it is so horrible! How do you do? Why?” (Female, 19, participant).* The same participant considered that the people who should be justifying themselves are the drinkers, not the people who decided not to drink.*“I should be the one asking them ‘Why do you need to drink?’” (Female, 19, non-drinker).*

Moreover, there would be a hierarchy of reasons and non-drinking youth would receive more or less remarks depending on the reason given. Religion seemed to be one of the most respected reasons. *“There are people who do not drink due to their religion and I totally respect that.” (Male, 15, drinker).*

Driving was another accepted reason for not drinking. Young drivers would even be encouraged not to drink. “*I would even say that we would go as far as encouraging someone not to drink if he is driving, whereas when you say you do not drink for another reason, it is a totally different reaction.” (Male, 18, drinker).*

Therefore, the reaction of a drinking peer would sometimes depend on the reason given for not drinking. For instance, a participant reported that if the reason was not validated by some peers, they may pressure non-drinkers to drink. It could even be that they exclude non-drinkers from future social events. “Y*ou have to justify (why you do not drink) and they have to accept the justification. If you say ‘Yeah no, I just do not feel like drinking today’, ‘I am not interested in that’, they will either try to force you, or they will make remarks or they will simply not invite you to other parties […].” (Female, 16, non-drinker).*

The pressure to drink would not necessarily come from peer comments, but also from the group effect and the resulting pressure to do as others do. *“It is true that when we go out, we often have the pressure, well given that the others often go out drinking, if we do not (drink), I think there will be a kind of pressure (to drink).” (Male, 20, non-drinker).*

However, some non-drinking participants reported that they felt no pressure to drink and perceived the questions they were asked as a positive interest in their non-drinking status. *“People do not care. I mean nobody ever insisted that I drink and even people in general are interested, but in a positive way, they say ‘Why don't you drink, what are the reasons? *etc*.’, there has never been anyone who has forced me to drink, nobody.” (Male, 18, non-drinker).*

### Non-drinkers strategies

Some youth presented strategies to hide their non-drinking status from others.

During parties, having a friend who does not drink either would help non-drinkers not feeling isolated during such events.*“(I do) not necessarily (feel excluded), I have a friend who does not drink either. So it is fine.” (Female, 20, non-drinker).*

Sometimes, some non-drinkers would use strategies to hide their non-drinking status. This would especially happen when they feel under pressure and/or are annoyed by comments from others. For instance, some participants considered that holding a glass was sufficient, regardless of whether its contents were alcoholic or not. They reported sometimes taking the glass they were handed without drinking, ant later on, give it to a drinking peer. “*Well, for me, the parties I have been to, you just have to have something in your glass, and then people do not look at what you put in your glass either, that is all.” (Male, 17, non-drinker).*

Non-alcoholic beverages were also considered by some young people as alternatives to alcoholic drinks. *“Alcohol-free beer can also be an option (alternative to alcohol).” (Female, 16, drinker).*

Some non-drinkers would simply say they are not drinking right now, not saying they do not drink at all. *“[…] If someone asks me, […] I do not say ‘I do not drink’. I would say ‘That is okay, I do not want to right now. I mean, like I am going to drink afterwards’.” (Female, 16, non-drinker).*

However, the majority considered such strategies unnecessary, especially if they were with friends who know they do not drink alcohol. *“I think beyond age and personality, it is precisely your group of friends that will determine whether or not you will have to use strategies […] If you are with a group of close friends […] you will need to use fewer strategies because it is generally accepted that you do not drink […].” (Female, 18, drinker).*

### Non-drinkers as resources

According to some participants, non-drinkers would sometimes automatically be the people who have to control the drinking of others. “*[…] Those who are not drunk are usually the ones who have to look after the rest of the group, somewhat against their will.” (Male, 19, drinker).*

This role could be uncomfortable for them and could prevent them from having a good time. For instance, one participant reported that she had spent the New Year’s Eve party hiding bottles of alcohol to prevent her friends from drinking more. “*[…] It is boring as a non-drinker to have to deal with people who drink alcohol. I mean, I spent my New Year's Eve locking bottles behind locked doors, taking the bottles out (of the hands), giving people water […].” (Female, 16, non-drinker).*

This responsibility could lead non-drinkers to feel guilty if they fail to intervene in a situation where help is needed, even though they have the ability to do so. “*As a sober person, if something happens, I would feel very bad […] because I was there and I had all my abilities to react, so I could be there and react if something happened, (for instance) hypothermia, someone choking or whatever, a stupid idea to jump off a bridge or lie down on a road.” (Male, 16, non-drinker).*

Having to play this role would prevent some young people from going to parties because they do not want to have that kind of responsibilities.*“[…] I have friends who have told me that it really pissed them off (to take care of their drinking-peers). So sometimes they do not come when they know it is a big party or if there will be a lot of alcohol. They say ‘Listen, it would have been nice to see you, but I would rather not because I do not want to take care of you tonight.” (Female, 18, drinker).*

On the other hand, a non-drinker considered that being a resource person could help non-drinkers forge links with other young people. *“Well, I think that you could be seen (as a non-drinker) as someone you can trust, someone to rely on if there is a problem […]. So, in a way, I think it also creates bonds between people.” (Male, 16, non-drinker).*

## Discussion

### Reasons for non-drinking

The main difference in terms of gender was the reason for not drinking alcohol for fear of sexual violences. According to literature, there is an association between hazardous alcohol use and sexual violence [34] and having experienced victimization may lead to a greater substance use [35, 36]. Contrary to our results, one study showed a correlation between fear of violence and greater alcohol use [37]. It is possible to hypothesize that, among drinkers, people who fear victimization consume more alcohol than the others. Based on our results, choosing not to drink might become a proactive strategy to manage the risk of sexual violence. In previous studies [38, 39], fear of sexual violence was also highlighted as a factor that might influence how young women consume alcohol, but more in terms of moderation or control rather than non-consumption. In this regard, avoiding drunkenness and keeping an eye on one's drink have been identified as part of the protective strategies employed by young women to produce a sense of safety and avoid men’s sexual violence in public spaces. Such findings have been discussed in light of the burden of responsibility in preventing violence and the fear of being blamed as a victim if intoxicated [38, 40, 41]. This self-protection has also been identified more broadly outside of bars and alcohol contexts, in public spaces and urban outdoor activities, with the adoption of various strategies by women to limit their behaviors and avoid violence [42].

### Friends and socialization

Experiences in terms of social life were mixed among non-drinkers. For some, not drinking did not impact their social life while others reported that they could feel excluded because they were not in the norm, which is drinking. This feeling was reported by other non-drinkers in literature [43, 44]. Furthermore, as it is considered as abnormal, young non-drinkers would constantly have to justify their non-consumption of alcohol, which could become a burden for some of them. This may lead them to avoid drinking contexts and feeling left out as previously reported [43]. This would particularly happen as when alcohol becomes increasingly normalised as young people get older, and even more so in university contexts where various events would often revolve around alcohol. Therefore, some participants felt that as they grew older, they perceived more comments and pressure from their peers, as alcohol became legal and increasingly normalised. Others, however, felt that the pressure decreased with age, thanks to the maturity that young people gained and the fact that it was easier to assert themselves as they grew older.

Despite the fact that some data [1–7, 19, 26] indicate that the percentage of young people not drinking alcohol has increased in the last years, when legal age is reached, consistent with previous research [45], young drinkers may feel as being a majority and social drinking would remain the norm [45].

### Drinking normalization

Due to drinking normalization, some non-drinkers avoid drinking environments or use strategies to hide their non-drinking status. Some participants reported avoiding events where all their peers are drinking, as reported in a qualitative study led among 18–25-year-olds in Australia [43]. Unlike other studies, no participant reported invoking health or sport to their drinking peers as strategies to explain their non-drinking status [43]. Some, on the contrary, hid their drinking status by pretending to drink or not saying that they did not want to drink. According to some authors [46, 47], the strategies used to hide non-drinking status reinforce the normalization of drinking and would lead to a vicious circle. Therefore, there is a need to empower young non-drinkers to assert themselves. A way to do this would be to value them by making their non-drinking status a strength. Indeed, not being influenced by the majority and not giving in to the norm could represent a form of courage. This may contribute to the deconstruction of the bias according to which drinkers are cool as opposed to non-drinkers. Indeed, as reported in a British qualitative study led among 11–13 and 16–17 year olds [48], drinkers are often described as cool people who like to have fun.

### Non-drinkers as resources

As reported in the British qualitative study led among 16–25 year-olds non-drinkers [44], they are often designated as the “lifesavers” during parties. This status may put them under pressure and lead them to stop attending parties. Some non-drinkers participants also indicated that they might feel guilty if they were not able to help drinking people and can sometimes be blamed for not preventing problems. This echoes a Danish study led among 18–19 year olds [45], in which a participant reported that he did not want to be the one who is perceived as boring and excessively responsible, using the term “grandma” to refer to the role of someone, even a drinker, who looks at someone else’s alcohol consumption. On the other side, in the same study, some participants reported that they may not feel legitimate to moderate their friends’ drinking if they are themselves drunk. It is important to raise awareness among young people of the pressure on non-drinkers which can be overwhelming, and that the responsibility should be shared among all young people if their friends need help.

## Strengths and limitations

To the best of our knowledge, no previous qualitative research has investigated non-consumption of alcohol among youth in Switzerland. Moreover, to obtain an overall picture of non-consumption of alcohol among youth, we included both non-drinkers and drinkers to explore experiences and opinions. This qualitative study could pave the way for more targeted interventions that take into account drinking and non-drinking status and include discussion on non-drinking among youth. However, some limitations need to be discussed. First, findings are based on self-reported narratives and social desirability bias in responses cannot be totally excluded. Second, we occasionally used the snowball method to recruit participants and this method could present the risk of having participants with similar opinions or experiences and characteristics. We had to use this method when we encountered difficulties in recruiting participants. This was, for example, the case for drinkers under the age of 16 (below the legal drinking age). Third, the scope of our study was restricted to French-speaking Switzerland and our recommendations are not necessarily generalizable to other populations.

## Conclusions

This study shows that non-drinkers would be a minority in their peer groups and would always have to justify their non-drinking. Moreover, they would sometimes be designated as resources who have to control their drinking-peers who need help. It is therefore important to normalize non-consumption of alcohol among youth and young adults, to be socially accepted and not questioned. A way to normalize alcohol non-consumption could be to make visible non-drinkers by including them in prevention interventions. On the one hand, this would raise awareness among young people about the fact that some of their peers make the choice not to drink and that all reasons must be respected without discussion. On the other hand, non-drinkers could share their experiences and express their feelings and needs to their drinking peers. This would allow young drinkers to better understand non-drinkers and better include them during parties. Finally, including non-drinkers in prevention efforts would also provide them with support and the opportunity to share experiences with other non-drinkers, helping to address their potential sense of isolation.". This would promote good social and mental health among young non-drinkers.

Then, to improve the social life of non-drinkers, it seems important to promote inclusive parties where drinking is not central and has no profound role. Finally, non-drinkers are often identified as the ones who will help others at a party. It is important to raise awareness among young people of this as the pressure put on non-drinkers can be overwhelming and that responsibility should be shared among all.

## Data Availability

The datasets generated by the survey research during and/or analyzed during the current study are available upon reasonable request in the repository https://doi.org/10.16909/dataset/37.

## References

[1] Larm P, et al. The increased trend of non-drinking in adolescence: the role of parental monitoring and attitudes toward offspring drinking. Drug Alcohol Rev. 2018;37(Suppl 1):S34-s41. 29473244 10.1111/dar.12682

[2] Lintonen T, Nevalainen J. Has the role of personal income in alcohol drinking among teenagers changed between 1983 and 2013: a series of nationally representative surveys in Finland. BMJ Open. 2017. 10.1136/bmjopen-2016-013994. 10.1136/bmjopen-2016-013994 PMC5775469 28416499

[3] Ng Fat L, Shelton N, Cable N. Investigating the growing trend of non-drinking among young people; analysis of repeated cross-sectional surveys in England 2005–2015. BMC Public Health. 2018;18(1):1090. 30301472 10.1186/s12889-018-5995-3 PMC6178254

[4] Livingston M. Trends in non-drinking among Australian adolescents. Addiction. 2014;109(6):922–9. 24717214 10.1111/add.12524

[5] Wicki M, F Labhart, and G Gmel, *Erklärungsansätze für die Abnahme des Alkoholkonsums bei Jugendlichen mit einer Betrachtung der Situation in der Schweiz.* Sucht Schweiz, 2019.

[6] Pavlidis A, Ojajarvi A, Bennett A. Young people and alcohol abstention: youth cultural practices and being a non-drinker in Finland and Australia. J Youth Stud. 2019;22(8):1101–16.

[7] Pape H, Rossow I, Brunborg GS. Adolescents drink less: how, who and why? a review of the recent research literature. Drug Alcohol Rev. 2018;37:S98–114. 29573020 10.1111/dar.12695

[8] Kraus L, Nociar A. ESPAD report 2015: results from the European school survey project on alcohol and other drugs. Portugal: European Monitoring Centre for Drugs and Drug Addiction; 2016.

[9] McCabe SE, et al. Assessment of changes in alcohol and marijuana abstinence, co-use, and use disorders among us young adults from 2002 to 2018. JAMA Pediatr. 2021;175(1):64–72. 33044552 10.1001/jamapediatrics.2020.3352 PMC7551219

[10] Jordan MD et al., *La consommation de substances psychoactives des 11 à 15 ans en Suisse – Situation en 2018 et évolutions depuis.* Addiction Suisse, 2019.

[11] Office fédéral de la statistique [Federal Statistical Office]. Consommation habituelle d'alcool [Usual alcohol consumption]. Tableaux standards XLSX [standard tables XLSX]. 2022. Available from: https://www.bfs.admin.ch/bfs/de/home/statistiken/gesundheit/erhebungen/sgb/ergebnissepublikationen.assetdetail.28725083.html.

[12] Whitaker V, et al. Young people’s explanations for the decline in youth drinking in England. BMC Public Health. 2023;23(1):402. 36850006 10.1186/s12889-022-14760-y PMC9972726

[13] Conroy D, de Visser RO. Benefits and drawbacks of social non-drinking identified by British university students. Drug Alcohol Rev. 2018;37:S89–97. 28940414 10.1111/dar.12610

[14] Pennay A, et al. “There’s a lot of stereotypes going on”: a cross-national qualitative analysis of the place of gender in declining youth drinking. Int J Drug Policy. 2022;108: 103827. 35985206 10.1016/j.drugpo.2022.103827 PMC7614950

[15] Törrönen J, et al. Why are young people drinking less than earlier? identifying and specifying social mechanisms with a pragmatist approach. Int J Drug Policy. 2019;64:13–20. 30544091 10.1016/j.drugpo.2018.12.001

[16] Törrönen J, et al. ‘Social health’, ‘physical health’, and well-being: analysing with bourdieusian concepts the interplay between the practices of heavy drinking and exercise among young people. Int J Drug Policy. 2021;91: 102825. 32593513 10.1016/j.drugpo.2020.102825

[17] Hoel S, et al. Adolescent alcohol use, psychological health, and social integration. Scandinavian J Public Health. 2004;32(5):361–7. 10.1080/14034940410027894 15513669

[18] Piacentini MG, Banister EN. Managing anti-consumption in an excessive drinking culture. J Bus Res. 2009;62(2):279–88.

[19] Chok L, Surís JC, Barrense-Dias Y. Non-consumption of alcohol: the adolescents’ point of view. Lausanne: Centre universitaire de médecine générale et santé publique; 2023.

[20] Herring R, Bayley M, Hurcombe R. “But no one told me it’s okay to not drink”: a qualitative study of young people who drink little or no alcohol. J Substance Use. 2014;19(1–2):95–102.

[21] Jané-Llopis E, et al. What is the current alcohol labelling practice in the WHO European Region and what are barriers and facilitators to development and implementation of alcohol labelling policy? Geneva: World Health Organization; 2020. 32598114

[22] Staub C, Siegrist M. How health warning labels on wine and vodka bottles influence perceived risk, rejection, and acceptance. BMC Public Health. 2022;22(1):157. 35073894 10.1186/s12889-022-12564-8 PMC8785573

[23] Raby R. Public selves, inequality, and interruptions: the creation of meaning in focus groups with teens. Int J Qual Methods. 2010;9(1):1–15.

[24] O’Brien BC, et al. Standards for reporting qualitative research: a synthesis of recommendations. Acad Med. 2014;89(9):1245–51. 24979285 10.1097/ACM.0000000000000388

[25] Tong A, Sainsbury P, Craig J. Consolidated criteria for reporting qualitative research (COREQ): a 32-item checklist for interviews and focus groups. Int J Qual Health Care. 2007;19(6):349–57. 17872937 10.1093/intqhc/mzm042

[26] Surís JC, et al. Alcohol use and misuse among young people What about abstainers? a quantitative approach. Lausanne: Unisanté Centre universitaire de médecine générale et santé publique; 2022.

[27] Dir AL, et al. Gender differences in risk factors for adolescent binge drinking and implications for intervention and prevention. Front Psychiatry. 2017. 10.3389/fpsyt.2017.00289. 10.3389/fpsyt.2017.00289 PMC5743668 29312017

[28] Wilsnack SC, Wilsnack RW. International gender and alcohol research: recent findings and future directions. Alcohol Res Health. 2002;26(4):245–50. 12875033 PMC6676686

[29] Heary CM, Hennessy E. The use of focus group interviews in pediatric health care research. J Pediatr Psychol. 2002;27(1):47–57. 11726679 10.1093/jpepsy/27.1.47

[30] Frith H. Focusing on sex: using focus groups in sex research. Sexualities. 2000;3(3):275–97.

[31] Clarke V, Braun V. Thematic analysis, in encyclopedia of critical psychology. Springer, New York: New York, NY; 2014.

[32] Braun V, Clarke V. Using thematic analysis in psychology. Qual Res Psychol. 2006;3(2):77–101.

[33] Hsieh H-F, Shannon SE. Three approaches to qualitative content analysis. Qual Health Res. 2005;15(9):1277–88. 16204405 10.1177/1049732305276687

[34] Burke L, et al. Alcohol, drug use and experiences of sexual violence victimisation among first-year college students in Ireland. J Sexual Aggress. 2023. 10.1080/13552600.2023.2216221.

[35] Pinchevsky GM, Fagan AA, Wright EM. Victimization experiences and adolescent substance use: does the type and degree of victimization matter? J Interpers Violence. 2014;29(2):299–319. 24144722 10.1177/0886260513505150 PMC4143976

[36] Earnshaw VA, et al. Peer victimization, depressive symptoms, and substance use: a longitudinal analysis. Pediatrics. 2017. 10.1542/peds.2016-3426. 10.1542/peds.2016-3426 PMC8918284 28562268

[37] Couture M-C, et al. Effect of fear of victimization on hazardous alcohol drinking, tobacco, and marijuana use among university students: a tale of two sexes. Addict Behav. 2020;106: 106355. 32088422 10.1016/j.addbeh.2020.106355

[38] Fileborn B. Doing gender, doing safety? young adults’ production of safety on a night out. Gend Place Cult. 2016;23(8):1107–20.

[39] Brooks O. Consuming alcohol in bars, pubs and clubs: a risky freedom for young women? Ann Leisure Res. 2008;11(3–4):331–50.

[40] Brooks O. ‘Guys! Stop doing it!’: young women’s adoption and rejection of safety advice when socializing in bars, pubs and clubs. Br J Criminol. 2011;51(4):635–51.

[41] Weiss KG. Too ashamed to report: deconstructing the shame of sexual victimization. Fem Criminol. 2010;5(3):286–310.

[42] Wesely JK, Gaarder E. The gendered “nature” of the urban outdoors: women negotiating fear of violence. Gend Soc. 2004;18(5):645–63.

[43] Hardcastle SJ, O’Connor M, Breen LJ. Exploration of young adults’ influences on, and consequences of, avoiding alcohol consumption. Subst Use Misuse. 2019;54(5):831–40. 30618328 10.1080/10826084.2018.1546744

[44] Herring R, Bayley M, Hurcombe R. “But no one told me it’s okay not to drink”: a qualitative study of young people who drink little or no alcohol. J Subst Use. 2014;19(1–2):95–102.

[45] Demant J, Järvinen M. Social capital as norms and resources: focus groups discussing alcohol. Addi Res Theory. 2011;19(2):91–101.

[46] Caluzzi G, et al. Declining drinking among adolescents: are we seeing a denormalisation of drinking and a normalisation of non-drinking? Addiction. 2022;117(5):1204–12. 34159676 10.1111/add.15611 PMC7614939

[47] Sznitman SR. Drug normalization and the case of Sweden. Contemp Drug Probl. 2008;35(2–3):447–80.

[48] Davies EL, Martin J, Foxcroft DR. Young people talking about alcohol: Focus groups exploring constructs in the prototype willingness model. Drugs Educ Prev Policy. 2013;20(4):269–77.

